# Acute promyelocytic leukemia with myelofibrosis: A case report and literature review

**DOI:** 10.1097/MD.0000000000024567

**Published:** 2021-04-02

**Authors:** Mengyu Xiao, Ling Qin, Xiaona Niu, Pan Zhou, Junwei Niu, Shengjie Wei, Dan Li, Liurui Dou, Wanjun Zhang, Lei Zhang, Kai Sun, Yanliang Bai

**Affiliations:** a Department of Hematology, Zhengzhou University People's Hospital; b Department of Hematology, First Affiliated Hospital, College of Clinical Medicine, Henan University of Science and Technology, Luoyang; c Department of Hematology, Institute of Hematology, Henan Provincial People's Hospital, Zhengzhou, Henan, PR China.

**Keywords:** acute promyelocytic leukemia, arsenic trioxide, case report, myelofibrosis, retinoic acid

## Abstract

**Rationale:**

Acute promyelocytic leukemia (APL) with myelofibrosis (MF) is rare, and only 14 cases have been reported in the literature to date.

**Patient concerns:**

A 42-year-old woman was admitted to the hospital with easy bruising and excessive bleeding. With the remission of the primary disease during treatment, the degree of fibrosis did not decrease, but worsened progressively.

**Diagnosis:**

The woman was diagnosed with acute promyelocytic leukemia with secondary myelofibrosis.

**Interventions:**

All-trans retinoic acid (ATRA) was discontinued after 6 months of complete remission of APL. Arsenic trioxide (ATO) was discontinued because of supraventricular tachycardia 9 months after complete remission of APL.

**Outcomes:**

After withdrawal of ATRA for 2 months, the degree of fibrosis was significantly alleviated, and after withdrawal of ATRA for 8 months and ATO for 5 months, bone marrow biopsy showed no reticular fiber deposition.

**Lessons:**

In this case report and review of an additional 14 cases of APL with MF, we highlighted the importance of the degree of MF to be evaluated by bone marrow biopsy at the time of bone marrow aspiration when APL is suspected. If MF is present, the type of MF should be determined in a timely manner, and appropriate intervention measures should be taken accordingly.

## 1 Introduction

JAK2V617, CALR, or MPL is usually positive in patients with primary myelofibrosis. Myelofibrosis (MF) is characterized by megakaryocytic proliferation and atypia.^[1]^ MF is more common in acute megakaryocytic leukemia (AML-M7),^[2]^ chronic myeloid leukemia (CML),^[3]^ and other diseases, and is a negative prognostic factor. However, only 14 cases of acute promyelocytic leukemia (APL) associated with myelofibrosis have been reported at home and abroad, and most of them are in the form of individual cases. In this paper, we present the clinical data of a patient with acute promyelocytic leukemia combined with myelofibrosis, with a review of relevant literature.

## 2 Case presentation

**Figure 1:**
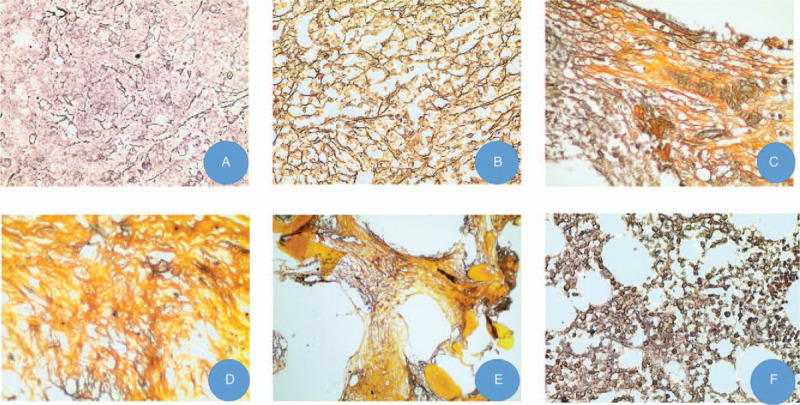
Result of Gomori staining in bone marrow biopsy of acute promyelocytic leukemia with myelofibrosis. A: Bone marrow biopsy at diagnosis shows a mild increase in reticulin fibers (grade 1) (Gomori stain, ×40). B: A repeated bone marrow biopsy at the relapse of APL shows a moderate increase in reticulin fibers (grade 2) (Gomori stain, ×40). C: Bone marrow biopsy image after achievement of complete remission shows considerable increase (grade 3) in bone marrow fiber content (Gomori stain, ×40). D, E: Bone marrow biopsy during maintenance therapy shows persistent extensive fibrous tissue deposition (grade 3) (Gomori stain, ×40). F: Bone marrow biopsy after withdrawal of ATRA for 8 months and ATO for 5 months showing recovery of normal hematopoiesis without myelofibrosis (Gomori stain, × 40). APL = acute promyelocytic leukemia, ATO = arsenic trioxide, ATRA = all-trans retinoic acid.

The patient was a 42-year-old woman who was admitted to a local hospital in September 2018, presenting with easy bruising and excessive bleeding. Routine blood examination in the outpatient department showed a leukocyte count of 1.43 × 10^9^/L, a hemoglobin level of 57 g/L, and a platelet count of 12 × 10^9^/L. Coagulation tests showed: prothrombin time (PT) of 14.12 seconds, activated partial thromboplastin time (APTT) of 24.50 seconds, and fibrinogen level of 0.86 g/L. The patient refused hospitalization. Half a day later, the patient again experienced gingival bleeding and was admitted to the local hospital. Physical examination on admission revealed anemia, scattered ecchymosis, and no enlargement was observed in the superficial lymph nodes. Sternal tenderness was negative, no abnormality in cardiopulmonary auscultation. Hepatomegaly or splenomegaly was not observed, and there was no edema in either lower extremity. Routine blood examination showed a leukocyte count of 4.90 × 10^9^/L, a hemoglobin level of 75 g/L, and a platelet count of 10 × 10^9^/L. Coagulation tests showed: prothrombin time (PT) of 14.62 seconds, activated partial thromboplastin time (APTT) of 23.30 seconds, and fibrinogen level of 0.77 g/L. Bone marrow aspirate showed polar hyperplasia, abnormal granulocytosis, and pathological promyelocytic granulocytes (70.5%). The morphological characteristics were large and uniform cell bodies, mostly round, quasi-circular, or elliptic. The cytoplasm was blue, abundant, and full of dense and thick azurophilic granules. The nuclei were irregular, mostly butterfly shaped, with 2 to 5 fine nucleoli. The proportion of erythrocytes was decreased, intermediate erythroblasts were occasionally observed, and the morphology was normal. Lymphocytes accounted for 4%, and their morphology was generally normal. Megakaryocytes were not observed in the whole film, and platelets were rare. Myeloperoxidase staining: most of the cells were strongly positive. Flow cytometry detection of bone marrow showed 83.21% of the abnormal nucleated cells expressing CD117, CD33, CD13, CD123, MPO, and CD9, weakly expressed CD64, but not CD38, CD14, CD34, HLA-DR, CD7, CD15, CD11b, CD22, CD19, CD5, CD10, CD20, CD36, CD4, TDT, CD56, cCD79a, and mCD3. Conclusion: Abnormal myeloid cells were found in the specimens, occupying 83.21% of the nuclear cells, which was consistent with the phenotype of AML. The leukemia cells were negative for CD34 and HLA-DR, with high SSC, considering APL with PML/RARA. Bone marrow biopsy showed hyperplasia (>90%), and the number of cells in the immature stage of the medullary system was significantly increased. Megakaryocytes were rare, with lobed nuclei. Reticular fiber staining (grade 1) (Fig. 1A). The fusion gene was positive for PML-RARA, positive for FLT3-ITD, negative for CEBPa-(bZIP), negative for NPM1, and negative for c-KIT (EXON17).

The patient was diagnosed as APL according to the results of bone marrow morphology, cytogenetics, and molecular biology. The patient was started on all-trans retinoic acid (ATRA) at 25 mg/m^2^ orally on days 1 to 14, arsenic trioxide (ATO) (0.16 mg/kg) intravenously on days 2 to 14, and daunorubicin at 40 mg/m^2^ intravenously on days 6 to 8. At the same time, continuous blood transfusion, infection control, and other symptomatic support treatment were given to the patient. On October 8, 2018 (the 14th day of chemotherapy), the blood routine showed a leukocyte count of 7.64 × 10^9^/L, a hemoglobin level of 65 g/L, and a platelet count of 54 × 10^9^/L. The patient was strongly requested to be discharged for personal reasons, which led to the interruption of treatment.

On March 27, 2019, she was admitted to our hospital with fever. Routine blood examination showed pancytopenia, with a leukocyte count of 2.01 × 10^9^/L, a hemoglobin level of 80 g/L, and a platelet count of 13 × 10^9^/L. Coagulation tests showed: prothrombin time (PT) of 9.7 seconds, activated partial thromboplastin time (APTT) of 23.50 seconds, fibrinogen level of 22.9 μg/mL, and D-dimer of 4.97 mg/L. Her ultrasound report showed a large spleen, 152 mm in length and 42 mm in thickness. Flow cytometry of bone marrow showed that 71.68% were abnormal myeloid immature cells expressing CD34, CD117, CD33, CD13, CD123, CD64, and MPO, partially expressing CD38, CD7, and cTdT, but did not express HLA-DR, CD15, CD11b, CD14, CD300e, CD10, CD19, CD20, CD4, CD8, CD3, CD2, CD56, cCD79a, cCD3, CD22, and CD5. Thus, AML with CD7 expression was considered. 47,XX,+8, t(15;17) (q24;q21) [16]/46, XX [4] for chromosome tests, and positive for PML-RARA. Screening for leukemia-mutated genes revealed that the FLT3-ITD generation mutation, ASXL1 positive, and RUNXI-positive. Bone marrow aspiration revealed hyperplasia, myeloblasts, and promyelocytic granulocytes accounting for 80%, with cell bodies of different sizes, round-like shape, round nuclei, and easy to see distortion and folding. Some azurophilic granules appeared in the cytoplasm. Cells at other stages were rare or absent. The hyperplasia of erythroid was inhibited, and only one naked megakaryocyte was found throughout the film. Bone marrow biopsy indicated moderate myelofibrosis (grade 2) (Fig. 1B).

Considering the patient's clinical manifestations, bone marrow morphology, chromosome, gene examination results, and previous medical history, the diagnosis of recurrence of acute promyelocytic leukemia and secondary myelofibrosis were made. ATO with 0.16 mg/kg on days 1 to 28 was administered immediately after admission, ATRA (with 25 mg/m^2^ on day 16 to 31), and idarubicin (IDA) (with 8 mg/m^2^ on days 2, 12, and 13). Blood transfusion and other supportive treatments were administered to prevent retinoic acid differentiation syndrome.

Bone marrow examination on May 7, 2019, showed complete morphology remission and was negative for PML-RARA and FLT3-ITD. ATRA (25 mg/m^2^ on days 2 to 19) and ATO (0.16 mg/kg on days 1 to 19) were administered. On June 7, 2019, repeated bone marrow aspiration showed dry tap at multiple sites, and the report indicated that the bone marrow morphology appeared normal. However, the bone marrow biopsy report revealed severe myelofibrosis in the third grade (Fig. 1C). Cytogenetics showed a normal karyotype (46, XX[11]). Ultrasound showed that her spleen had recovered to a normal size. The molecular test for PML-RARA was consistently negative. She was then administered idarubicin (8 mg/m^2^ on days 1 to 3) and cytarabine (100 mg/m^2^ on days 1 to 6) from June 26, 2019. A month later, she was treated with ATRA (25 mg/m^2^ on days 1 to 14) and ATO (0.16 mg/kg on days 1 to 14). On September 11, 2019, bone marrow aspiration was repeated, and the bone marrow biopsy report showed diffuse reticulin deposition (Grade 3) (Fig. 1D).

From September 14 to October 10, ATRA was performed daily. After that, she regularly took ATRA and compound realgar natural indigo tablets (CRNIT) (the oral agent of ATO) from the hospital. Bone marrow biopsy was performed on November 14, 2019 (grade 3) (Fig. 1E). Considering that ATRA may lead to myelofibrosis, ATRA was discontinued from November 16, 2019, and only CRNIT was administered for maintenance treatment. On January 16, 2020, the patient was admitted for bone marrow trephine, which showed resolution of myelofibrosis (grade 0), and the MPN-related genes JAK2, CALR, and MPL were negative. From February 3 to February 17, 2020, the patient was treated with CRNIT alone. The patient had obvious palpitation, and the ECG indicated supraventricular tachycardia, which was considered to be an adverse reaction to ATO. Therefore, CRNIT was discontinued on February 18, 2020, and drug therapy for heart rhythm control was administered. Bone marrow aspiration and biopsy were performed again on July 6, 2020, which showed consistent complete morphological remission and disappearance of reticulin (Grade 0) (Fig. 1F). During the last clinical review in July 2020, the patient was clinically good. Her blood counts, coagulation assay results, and spleen size were consistently normal.

## 3 Discussion and literatures review

In AML-M7,^[2]^ CML,^[3]^ myelodysplastic syndrome (MDS),^[4]^ and other diseases, MF of different degrees is not uncommon and is associated with poor prognosis. However, MF is extremely rare in APL, and only 14 cases have been reported. In this case, 14 domestic and foreign patients with APL, 14 patients achieved molecular or morphological remission, and 1 patient achieved partial remission in morphology after induction remission. According to this study, it is shown that in APL, the combination of MF does not affect the therapeutic effect and prognosis of the primary disease.

Fourteen cases of acute promyelocytic leukemia with myelofibrosis have been reported in the literature (from 1991 to 2019)^[5–16]^ at home and abroad. In order to clarify the clinical and laboratory characteristics of APL patients with MF, combined with this case, the relevant characteristics were summarized as shown in Table 1. MF was mostly found in middle-aged and elderly people, whereas APL was mostly found in young and middle-aged people. The median age at diagnosis was 34 years (range, 18–83 years). Compared with APL patients without MF, APL patients with MF mainly showed similar symptoms, such as bleeding, anemia, infection, and abnormal coagulation function. There were 7 cases with abnormal coagulation function, 3 cases with normal coagulation function, and 5 cases without relevant data. There were 2 cases of primary myelofibrosis, 7 cases of reactive myelofibrosis, 4 cases of treatment-related myelofibrosis, and 2 cases could not be determined due to the absence of follow-up data from bone marrow biopsy. There were 5 cases with CD34 or HLA-DR positivity, 5 cases with negative CD34, and 5 cases without relevant data. Expression of CD34 or HLA-DR was not associated with aggravation of fibrosis. After induced remission treatment, 1 patient achieved partial remission in morphology, and the remaining 14 patients achieved molecular or morphological remission.

**Table 1:** Clinical features of this case along with 14 patients of acute promyelocytic leukemia with myelofibrosis reported in the literature.

Case	Sex/age, y	Presentation and blood routine	Coagulation function	The size of spleen	Morphology	Dry tap	Molecular biology	CD34, HLA-DR	Treatment	Response	The outcome of fibrosis	Follow-up
Our case	F/42	Easy bruising, bleeding, pancytopenia	Severe abnormal	Impalpable	Blasts, MPO strongly positive, grade 1 myelofibrosis	No	JAK2, CALR, MPL negative; FLT3/ITD, ASXL1, RUNXI positive	No	ATRA+ATO+DNR/IDA	CR	Aggravation	>20 mo
^[4]^	F/22	Fever, easy bruising, anemia, thrombocytopenia	Normal	NA	Blasts, MPO positive, increased reticulin	Yes	NA	Yes	ATRA+chemotherapy	CR	NA	>1 y
^[5]^	M/26	DIC, fever, anemia, thrombocytopenia	Severe abnormal	NA	Blasts, faggot cell, severe and diffuse fibrosis	Yes	NA	NA	ATRA+DNR+BHAC	CR	Alleviation	Relapse 2 years later, then lost of follow-up
^[6]^	M/18	DIC, anemia, thrombocytopenia, leukocytosis	Severe abnormal	Moderate splenomegaly	Blasts, MPO strongly positive, diffuse fibrosis	Yes	NA	Yes	ATRA+DNR+BHAC+6-MP	CR	Alleviation	>21 mo
^[7]^	M/25	Bleeding, sternal tenderness, anemia, thrombocytopenia	Mildly abnormal	Normal	Blasts, MPO strongly positive, diffuse fibrosis	NA	NA	NA	ATRA+DNR	CR	Persistent	NA
^[8]^	F/34	Fever, bleeding, pancytopenia	Severe abnormal	NA	Blasts, severe reticulin fibrosis	Yes	NA	Yes	ATRA+ATO	CR	Alleviation	NA
^[9]^	M/48	Fatigue, pancytopenia	NA	Impalpable	Blasts with Auer rods, MPO positive, tear-drop shaped erythrocytes, severe reticulin fibrosis	Yes	NA	Yes	ATRA+IDA	CR	Persistent	>4 y
^[10]^	F/83	Fatigue, pancytopenia	NA	Splenomegaly	Blasts, megakaryocytes hyperplasia, grade 3 myelofibrosis	NA	JAK2 V617, FLT3 D835 positive	NA	ATRA+ATO	CR	Alleviation	NA
^[11–1]^	F/28	Palpitation, headache, dizziness, anemia, leukopenia	NA	Impalpable	Blasts with Auer rods, faggot cell, MPO positive, increased reticulin	NA	negative	No	ATRA+ATO	CR	Aggravation	>2 mo
^[11–2]^	M/24	Bleeding, easy bruising, anemia, thrombocytopenia, leukocytosis	Mildly abnormal	NA	Blasts with Auer rods, faggot cell, severe reticulin fibrosis	NA	FLT3 D835 positive	No	ATRA+IDA	CR	Alleviation	Relapse 15 mo later, then lost of follow-up
^[11–3]^	F/42	Easy bruising, menorrhagia, pancytipenia	NA	NA	Blasts with Auer rods, grade 2 myelofibrosis	Yes	NA	Yes	ATRA+ATO	CR	NA	>1 y
^[12]^	F/53	High fever, cellulitis of right lower limb, pancytopenia	Normal	NA	Blasts, MPO positive, increased reticulin	Yes	negative	No	ATRA+IDA	CR	Alleviation	>16 mo
^[13]^	F/62	Weight loss, hepatosplenomegaly, anemia, thrombocytopenia	NA	Splenomegaly	Blasts, tear-drop shaped erythrocytes, grade 1 myelofibrosis	NA	JAK2 V617, SF3B1 positive	NA	ATRA+DNR	CR	persistent	NA
^[14]^	F/34	Purpura of lower limbs, pancytopenia	Severe abnormal	Normal	Blasts with Auer rods, faggot cell, MPO strongly positive,mild fibrosis	NA	NA	No	ATRA+chemotherapy	CR	Aggravation	Died of septic shock after the third course of consolidation chemotherapy
^[15]^	F/44	Bleeding, fatigue, anemia, thrombocytopenia, leukocytosis	Normal	Normal	Blasts, MPO positive	No	NA	NA	ATO	PR	Dry tap appears on the 45th day of induction therapy, grade 3 myelofibrosis	ATRA and DNR

6-MP = 6-mercaptopurine, ATO = arsenic trioxide, ATRA = all-trans retinoic acid, BHAC = enocitabine, CR = complete remission, DIC = disseminated intravascular coagulopathy, DNR = daunorubicin, F = female, HHT = homoharringtonine, IDA = idarubicin, M = male, MF = myelofibrosis, NA = not available.

Kwong et al^[14]^ reported a case of acute promyelocytic leukemia with primary myelofibrosis (PMF). The patient presented with JAK2 V617F gene mutation and hepatosplenomegaly, which met the diagnosis of PMF and responded well to ATRA and ATO. Chronic inflammatory diseases, hair cell leukemia, other lymphocytic tumors, and toxic (chronic) bone marrow diseases can all cause reactive myelofibrosis.^[17]^ Islam reviewed the clinical features of 34 patients with acute myeloid leukemia, about one-third (12/34) of whom had various degrees of bone marrow fibrosis at the first diagnosis of AML. Bone marrow fibrosis can be reversed after effective anti-leukemia therapy and has no effect on whether complete remission of the primary disease can be achieved. In addition, fibrosis did not affect regeneration of the hematopoietic system.^[18]^ However, in our case, the patients’ MPN-related genes (such as JAK2, CALR, and MPL) were negative, and there were no obvious hepatosplenomegaly or other medullary hematopoiesis. Only mild bone marrow fibrosis was observed. In the case of APL illness is aggravating, bone marrow fibrosis was not parallel with the primary disease. Therefore, there is no sufficient basis for the diagnosis of PMF or reactive myelofibrosis,^[1]^ which is considered to be treatment-related MF.

**Figure 2:**
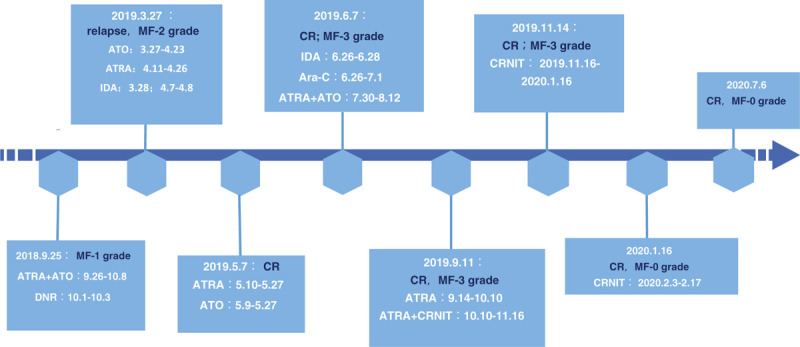
Timeline of treatment for acute promyelocytic leukemia with myelofibrosis. Note. Ara-C = cytarabine, ATO = arsenic trioxide, ATRA = all-trans retinoic acid, CR = complete remission, CRNIT = compound realgar natural indigo tablets (oral arsenic agent), DNR = daunorubicin, IDA = idarubicin, MF = myelofibrosis.

Acute promyelocytic leukemia (APL) is a disease with a good prognosis. With the standardized application of all-trans retinoic acid (ATRA) and arsenic (ATO), complete remission and cure rates for this disease have been further improved. However, it has been reported that both ATRA and ATO may cause myelofibrosis during treatment. Venkatesan reported a patient with APL who was treated with ATO alone. Bone marrow puncture was performed at the initial diagnosis, but on the 45th day after ATO treatment, the repeated bone marrow puncture showed obvious dry tap, and the biopsy results indicated severe myelofibrosis (grade 3).^[16]^ Hatake observed 13 patients with APL, but without MF at the initial diagnosis. After ATRA treatment, 11 of them had collagen fibers in their bone marrow, and MF was relieved to varying degrees after ATRA withdrawal or consolidation chemotherapy. It was also found that an extremely low concentration of retinoic acid can stimulate the growth of collagen fibers in vitro.^[19]^ In primary myelofibrosis models, oral active retinoic acid receptor antagonists may restore normal platelet production and bone integrity and significantly reduce fibrosis.^[20]^ In this study, 14 domestic and foreign patients were treated with ATRA combined with chemotherapy or ATO alone, and 2 patients had JAK2 V617 gene mutations, which supported the diagnosis of PMF. Seven patients with APL showed MF at the initial diagnosis, and the degree of MF was reduced or unchanged at the remission stage of APL, which was considered a reactive MF. Three patients with aggravated MF were considered to have treatment-related MF. Two cases could not be determined due to the absence of follow-up data from the bone marrow biopsy. In this case, the degree of MF was gradually aggravated during the treatment, and a reexamination of the bone marrow biopsy showed disappearance of reticulin 2 months after the discontinuation of ATRA. Eight months after ATRA withdrawal, and 5 months after CRNIT, repeated bone marrow biopsy showed no evidence of myelofibrosis (Fig. 2), which supported the diagnosis of treatment-related bone marrow fibrosis. This indicates that myelofibrosis in APL patients is heterogeneous, and more cases need to be accumulated to be accurately stratified.

In this study, we summarized our case and 14 cases at home and abroad, and found that the therapeutic effect of APL with MF was not significantly different from that of patients without MF. However, the underlying mechanism remains to be explored. MF is associated with the involvement of a series of cytokines, such as transforming growth factor (TGF-β),^[7]^ platelet-derived growth factor (PDGF),^[21]^ and basic fibroblast growth factor (bFGF).^[22]^ Megakaryoblasts can produce TGF-β, and myelofibrosis is more common in acute megakaryocytic leukemia. Terui et al^[2]^ proposed that the culture medium of megakaryoblasts could stimulate the growth of collagen fibers better than that of other types of leukemia. Megakaryoblasts can produce and secrete activated TGF-β and stimulate collagen synthesis. Among the 14 cases reported at home and abroad, one case of bone marrow biopsy report showed hyperplasia in megakaryocytes, while the remaining 13 cases did not report whether megakaryocytes were in hyperplasia. In our case, multiple bone marrow aspiration and biopsy reexamination at the later stage showed that the proliferation of megakaryocytes was normal. The mechanism of myelofibrosis in the absence of active megakaryocyte proliferation needs to be further explored by accumulating a large amount of clinical data.

In addition to megakarocytes, leukemia cells can also produce transforming growth factor (TGF-β), platelet-derived growth factor (PDGF), and basic fibroblast growth factor (bFGF).^[7]^ CD34 and HLA-DR are markers of early hematopoietic stem progenitor cells, which are not or weakly expressed in most APL.^[17]^ Mori et al^[6]^ detected TGF-β overexpression in leukemia cells of APL patients with myelofibrosis using RT-PCR. MF appeared to be more common in APL-expressing CD34 and HLA-DR than in APL lacking these immature markers.^[7]^ We summarized the expression of CD34 or HLA-DR in the 15 cases, among which, 5 cases had different degrees of CD34 or HLA-DR expression, 5 cases had negative CD34 and HLA-DR expression, and 5 cases had no flow cytometric detection results, possibly due to dry extraction during bone marrow puncture. In our case, the results of flow cytometry at the initial diagnosis were negative for CD34 and HLA-DR, with mild myelofibrosis (grade 1). However, when the APL relapsed later, the expression of CD34 was detected, but without the expression of HLA-DR, with grade 2 myelofibrosis. Patients with APL who were tested for the expression of CD34 and HLA-DR were more likely to develop myelofibrosis than those who lacked expression of these immature markers. Therefore, we proposed that whether CD34-and HLA-DR-positive leukemia cells in the bone marrow of APL patients could predict the incidence of secondary myelofibrosis, which should be further explored in future studies.

FLT3-ITD is a marker of poor prognosis in acute myeloid leukemia. FLT3-ITD mutations consistently activate the activity of receptor tyrosine kinase and its downstream signaling pathway, leading to cell proliferation disorders and ultimately to treatment failure or disease recurrence.^[23–25]^ Epigenetic modifications such as ASXL1, DNMT3A, TET2, IDH1, and IDH2 can coexist with PML-RARα fusion genes, and these genes are associated with poor prognosis in disease-free survival.^[26]^ Another study showed that the use of ATRA and ATO inhibited the expression of FLT3-ITD.^[27]^ In our case, FLT3-ITD was positive at the initial diagnosis and recurrence of APL, but it was negative after induction therapy with ATRA and ATO, which supports the finding that the use of ATRA and ATO can suppress the expression of FLT3-ITD.

There are still some deficiencies in this study. First, due to the rarity of APL cases with MF and the retrospective nature of the study, the sample size is small. Second, the follow-up survival time and the degree of remission of MF are far from sufficient. Third, there may have been a selection bias. Bone marrow biopsy is not a routine examination in APL, and some patients may have subsequent MF, thus missing the best time to observe and take measures. Therefore, a large number of multicenter, prospective studies are needed to describe the outcome, clinical diagnosis, and treatment of myelofibrosis in APL.

APL with MF is an uncommon clinical case. The clinical manifestations and treatment responses of APL with MF were similar to those of APL without MF. In addition, some patients with APL were initially diagnosed with mild MF without dry tap, but the level of MF increased at a later stage. Therefore, we suggest that when APL is suspected, bone marrow biopsy should be performed at the same time to evaluate the degree of MF. If MF is present, the type of bone marrow fibrosis should be determined and appropriate intervention measures should be taken accordingly.

## 4 Conclusions

We suggest that when APL is suspected, the degree of MF should be evaluated by bone marrow biopsy at the time of bone marrow aspiration. If MF is present, the type of MF should be determined in a timely manner, and appropriate intervention measures should be taken accordingly. At the same time, we proposed to detect CD34- and HLA-DR-positive leukemia cells in the bone marrow of APL patients to predict the occurrence of MF, which should be further explored in future studies.

## Acknowledgments

The authors would like to thank Huihui Guo for her guidance in data collection and data analysis.

## Author contributions

**Conceptualization:** Mengyu Xiao, Ling Qin, Yanliang Bai.

**Data curation:** Xiaona Niu, Pan Zhou.

**Formal analysis:** Junwei Niu, Mengyu Xiao.

**Funding acquisition:** Kai Sun.

**Investigation:** Shengjie Wei, Dan Li, Liurui Dou.

**Methodology:** Mengyu Xiao, Kai Sun, Yanliang Bai.

**Validation:** Wanjun Zhang, Lei Zhang.

**Writing – original draft:** Mengyu Xiao, Ling Qin, Yanliang Bai.

**Writing – review & editing:** Mengyu Xiao, Kai Sun, Yanliang Bai.
